# Chronic infective arthritis with osteomyelitis of the ankle due to *Pseudomonas aeruginosa* infection in a middle-age woman: A rare causative pathogen requiring vigilance

**DOI:** 10.1097/MD.0000000000037344

**Published:** 2024-03-08

**Authors:** Chien-Liang Chen, Tai-Kuang Chao, Fu-Chiang Yeh, Ching-Lan Wu, Ching-Hsun Wang

**Affiliations:** a Division of Infectious Diseases and Tropical Medicine, Department of Internal Medicine, Tri-Service General Hospital, National Defense Medical Center, Taipei, Taiwan; b Department of Pathology, Tri-Service General Hospital, National Defense Medical Center, Taipei, Taiwan; c Division of Rheumatology, Immunology and Allergy, Department of Internal Medicine, Tri-Service General Hospital, National Defense Medical Center, Taipei, Taiwan; d Department of Radiology, Taipei Veterans General Hospital, Taipei, Taiwan.

**Keywords:** 6 months, case report, chronic arthritis, chronic synovitis, infective arthritis, *P aeruginosa*, septic arthritis

## Abstract

**Rationale:**

*Pseudomonas aeruginosa*-induced septic arthritis is a relatively uncommon phenomenon. It has been documented in children with traumatic wounds, young adults with a history of intravenous drug use, and elderly patients with recent urinary tract infections or surgical procedures.

**Patient concerns:**

Fifty-nine year-old female had no reported risk factors. The patient sought medical attention due to a 6-month history of persistent pain and swelling in her right ankle.

**Diagnoses:**

Magnetic resonance imaging and a 3-phase bone scan revealed findings suggestive of infectious arthritis with concurrent osteomyelitis. Histopathological examination of the synovium suggested chronic synovitis, and synovial tissue culture confirmed the presence of *P aeruginosa.*

**Intervention:**

Arthroscopic synovectomy and debridement, followed by 6 weeks of targeted antibiotic therapy for *P aeruginosa*.

**Outcomes:**

Following treatment, the patient experienced successful recovery with no symptom recurrence, although she retained a mild limitation in the range of motion of her ankle.

**Lessons:**

To our knowledge, this is the first reported case of chronic arthritis and osteomyelitis caused by *P aeruginosa* in a patient without conventional risk factors. This serves as a crucial reminder for clinicians to consider rare causative organisms in patients with chronic arthritis. Targeted therapy is imperative for preventing further irreversible bone damage and long-term morbidity.

## 1. Introduction

Infective arthritis caused by Gram-negative bacteria is relatively uncommon and accounts for approximately 10% to 20% of infected cases according to previous studies.^[1,2]^ *Pseudomonas aeruginosa*, a nonfermenting Gram-negative bacterium, is considered one of the possible causative pathogens contributing to septic arthritis. Although rarely reported, it has been documented causing septic arthritis in children following a traumatic wound or in young adults with intravenous drug use.^[3,4]^ Septic arthritis due to *P aeruginosa* often occurs in elderly patients with recent urinary tract infections and/or those who have undergone surgery.^[5,6]^ The present report documents a case involving a middle-age female with diabetes, without reported traditional risk factors, who was admitted for prolonged ankle pain and was finally diagnosed with chronic *P aeruginosa*-related ankle arthritis with osteomyelitis.

## 2. Case report

The patient signed the consent for participation in this case report after being fully informed about its purpose, implications, and the use of their anonymous medical data for educational and research purposes. The Ethics Committee of the Tri-Service General Hospital, National Defense Medical Center, approved the study (TSGHIRB No.: A202315174).

**Figure 1.**
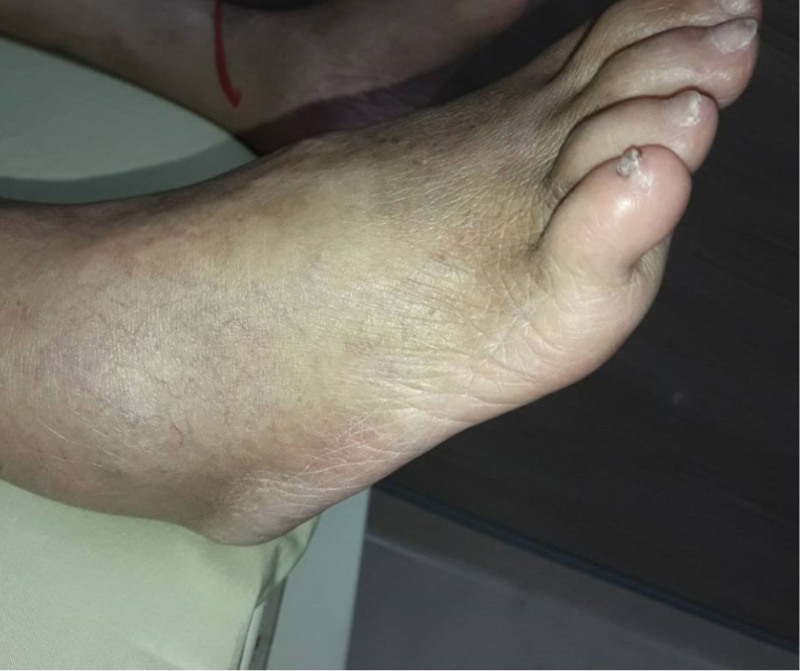
On admission, noticeable swelling was detected in the patient’s right ankle.

**Figure 2.**
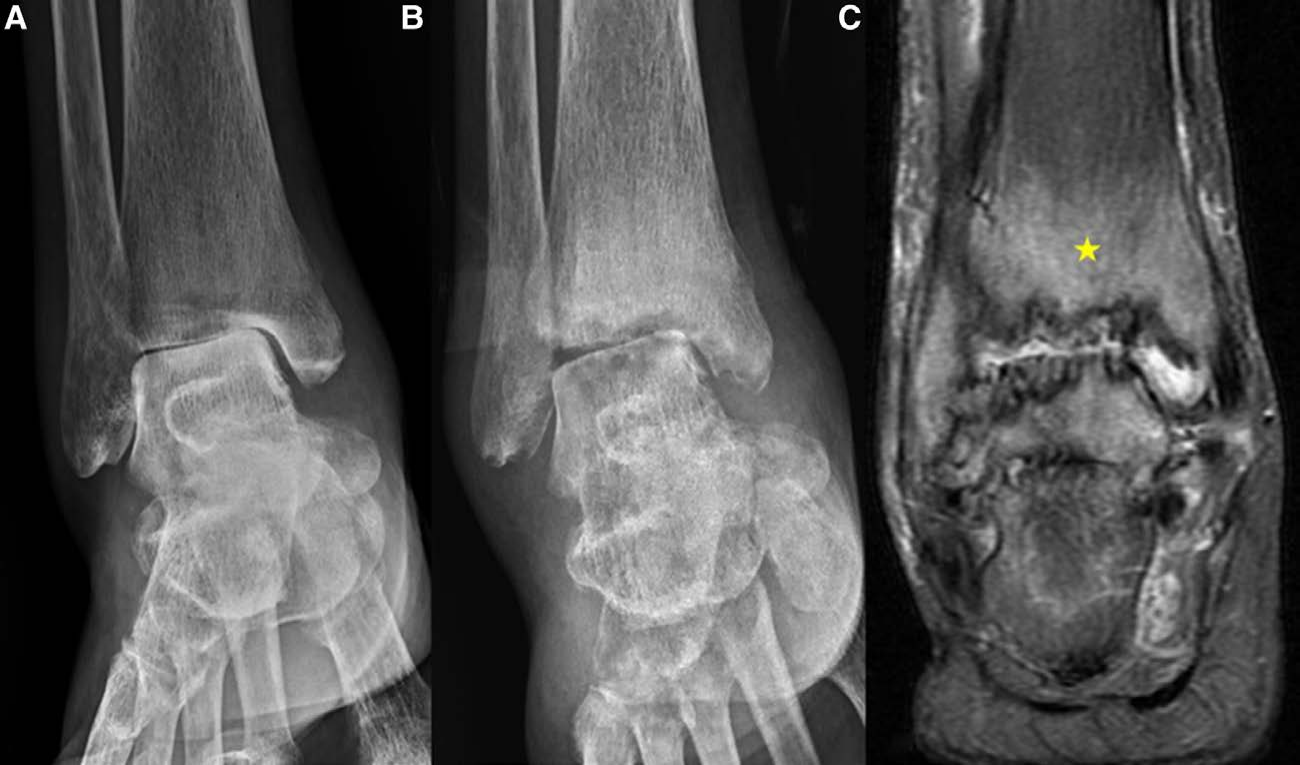
A series of image examinations including ankle radiography and magnetic resonance imaging in presented case: (A) The right ankle exhibited asymmetric joint space narrowing and minimal marginal osteophytosis during the initial admission. (B) Six months later, upon the second admission, new findings included subchondral bone erosion, pseudo-widened joint space, and surrounding soft tissue swelling. (C) T2-weighted imaging with fat suppression revealing bone erosion, surrounding bone marrow edema (star), small joint effusion, and conspicuous irregular synovial thickening.

**Figure 3.**
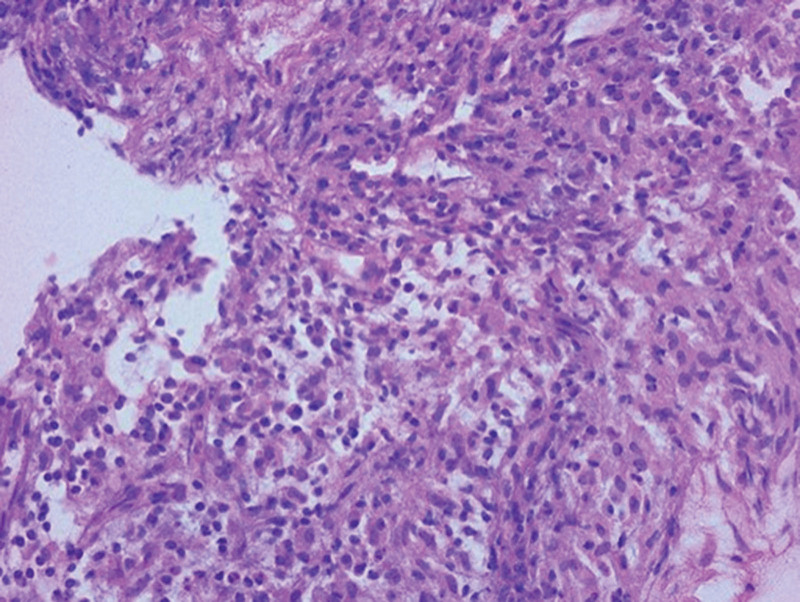
Histopathological examination revealing infiltrating lymphocytes and plasma cells, along with small vessel proliferation, indicating granulation tissue formation (hematoxylin and eosin stain, original magnification, ×400).

A 59-year-old female presented to the authors’ emergency department with a 7-day history of worsening pain and swelling in her ankles. She had a relatively recent admission record to the hospital due to cellulitis of the right foot approximately 7 months previously. During admission, intravenous antibiotic with cefazolin (1 g every 8 h) was initially administered; however, the condition did not improve, and redness and swelling extended to the right ankle. No visible wound was observed and ultrasonography of the ankle revealed synovial hypertrophy with only minimal fluid accumulation. Therefore, wound culture or synovial fluid culture via arthrocentesis for pathogen identification was not performed; moreover, blood culture results were negative. Radiography of the ankle revealed narrowing of the joint space of the right tibiotalar joint with subchondral sclerosis. Computed tomographic angiography of the lower limbs revealed no vascular disease. After switching the intravenous antibiotic to tigecycline (50 mg twice daily), her condition partially improved, and she was discharged from hospital 14 days after tigecycline treatment and continued with moxifloxacin 400 mg (once daily) for the ensuing 7 days. After a complete antibiotic treatment course, however, persistent swelling and pain in the ankle were still noted during the post-discharge follow-up at the infection outpatient department. Nonsteroidal anti-inflammatory drugs were prescribed but the symptoms persisted. Owing to worsening ankle pain and swelling, she attended emergency department 6 months after the last discharge. The patient denied intravenous drug use or a history of smoking, with no recent history of surgery or trauma. She had been diagnosed with type 2 diabetes mellitus 5 years previously, with oral hypoglycemic control (glimepiride, 1 mg once daily). The most recent glycated hemoglobin level measured 3 months before admission was 6.4%. On examination, the patient (height, 157 cm; weight, 42 kg; body mass index, 17 kg/m^2^) was alert and oriented, with a blood pressure of 120/70 mm Hg, heart rate of 101 beats/min, and body temperature of 36.6°C. Her skin exhibited mild erythema around the right ankle region and no visible skin wounds on the right lower limb. The ankle was swollen and stiff and she experienced severe pain during movement (Fig. 1). Pain score, assessed according to a visual analog scale, was 7/10. All other clinical examination results (heart, respiratory system, nervous system, and abdomen) were unremarkable. Results of laboratory investigations revealed a white blood cell count of 10.1 × 10^3^/μL, with 78% neutrophils (reference range 4.5–11.0 × 10^3^/mL), a C-reactive protein level of 1.62 mg/dL (reference range < 1 mg/dL), and a procalcitonin level of 0.02 mg/dL (reference range < 0.05 mg/dL). A high erythrocyte sedimentation rate (ESR; 98 mm/h) was observed (reference range, 0–20.0 mm/h). Rheumatoid factor and anticyclic citrullinated peptide antibody test results were negative. After admission, she was administered an empirical antibiotic (tigecycline, 50 mg twice daily). A series of the right ankle X-rays revealed newly developed subchondral erosions, indicating a possible deep-seated infection (Fig. 2A and B). Additionally, magnetic resonance imaging revealed extensive bone marrow edema in the right distal tibia and talus, as well as marked joint effusion and synovial thickening in the tibiotalar joint (Fig. 2C). Three-phase, technetium 99m bone scintigraphy revealed marked accumulation of radioactivity in the right ankle region. Septic arthritis with osteomyelitis was suspected and the patient then underwent arthroscopic synovectomy and debridement. Synovial biopsy and culture were performed during surgery. Synovial tissue culture revealed *P aeruginosa*. Biopsy revealed histological evidence of chronic synovitis (Fig. 3). Based on these clinical features, imaging findings, and microbiological and histopathological results, the patient was diagnosed with chronic infective arthritis complicated by osteomyelitis of the ankle due to *P aeruginosa* infection. Antibiotics were changed to ciprofloxacin (400 mg) every 8 h according to the antimicrobial susceptibility results. The patient improved gradually and was discharged on day 30 after 14-day intravenous ciprofloxacin and oral ciprofloxacin (750 mg twice per day) was continued and prescribed for an additional 4 weeks. Her ESR and C-reactive protein levels normalized, and the patient improved significantly with residual mild limitation of range of motion of the right ankle and no recurrence of symptoms reported at the 1-year follow-up.

## 3. Discussion

Septic arthritis is a dangerous and destructive articular disease that is associated with significant morbidity.^[7]^ Immediate diagnosis with appropriate treatment is crucial to prevent irreversible destruction and dysfunction of the joint. Gram-positive organisms, such as *Staphylococcus aureus* and *Streptococci* are major pathogens attributed to septic arthritis.^[8]^ Gram-negative bacterial arthritis has rarely been described in literature, and *Enterobacterales* species are the most frequently isolated pathogens.^[1,9]^ Risk factors for Gram-negative bacterial arthritis include concurrent bacteriuria, prosthetic joint or hip joint involvement, and immunosuppressive conditions such as diabetes, liver cirrhosis, and steroid use.^[1,2,10]^ The prognosis is poorer in patients with infective arthritis caused by Gram-negative bacteria than in those caused by Gram-positive organisms.^[9]^ This may be due to the delayed use of appropriately targeted antibiotics against Gram-negative bacteria. *P aeruginosa*, a nonfermentative Gram-negative bacterium, is also a potential pathogen in bacterial arthritis. Earlier reports have described *P aeruginosa* in children with infectious bacterial arthritis following open injuries or illicit drug use, such as heroin, in young adults.^[4]^ Recent studies have revealed that the elderly population may be more vulnerable to bacterial arthritis by *P aeruginosa.*^[6,11]^ Moreover, *P aeruginosa* has also been reported to cause infective arthritis in immunocompetent pregnant patients without any reported risk factors.^[12]^ With regard to our case, no predisposing conditions were reported for Gram-negative bacteria or *P aeruginosa* bacterial arthritis. Diabetes mellitus with optimal glycemic control (latest glycated hemoglobin, 6.4%) was the only underlying comorbidity. Therefore, antibiotics administered at the beginning were targeted at Gram-positive organisms until the final synovial tissue culture reports revealed *P aeruginosa* infection. Due to the initial delayed targeted antibiotic treatment for *P aeruginosa* causing persistent infection and inflammation of the joint, we observed clinical features of chronic arthritis in the present case; more specifically, indolent clinical presentation on admission (prolonged ankle pain with only mild erythematous change around the ankle region, no fever, normal leukocyte count and procalcitonin level, near normal C-reactive protein level, but high ESR), destructive bone changes on follow-up radiography of the ankle, and three-phase positivity in a bone scan indicating concomitant osteomyelitis. Moreover, granulation changes in the synovial tissue suggested chronic synovitis, which is also compatible with the clinical presentation of chronic arthritis. Chronic synovitis associated with arthritis is often present in children with hemophilia, autoimmune or crystal-induced arthritis, and infective arthritis caused by tuberculosis or fungi.^[13]^ To our knowledge, this is the first reported case of chronic synovitis caused by *P aeruginosa.* Although no grossly visible wounds were initially observed, the most likely source of infection was the patient’s recent cellulitis. Such skin and soft tissue infections may cause an internal breach in the microvasculature that permits entry of the causative pathogen into the joint space, contributing to subsequent bacterial arthritis and osteomyelitis.^[13]^ Therefore, clinicians should consider rare causative organisms in patients with chronic arthritis. Immediate targeted therapy combined with surgical intervention may prevent irreversible bone destruction and long-term morbidity.

## 4. Conclusion

We present a case involving a middle-age woman who presented with persistent ankle swelling and pain, who was diagnosed with chronic *P aeruginosa* ankle arthritis and osteomyelitis. *P aeruginosa* should be considered as a causative pathogen in patients with infective arthritis. A history of previous skin infection is not a traditional risk factor but may, nevertheless, lead to such an infection.

## Author contributions

**Data curation:** Tai-Kuang Chao.

**Supervision:** Fu-Chiang Yeh.

**Validation:** Ching-Lan Wu.

**Writing – original draft:** Chien-Liang Chen.

**Writing – review & editing:** Ching-Hsun Wang.
